# The Significance of the Effect of Visceral Adiposity on Left Ventricular Diastolic Function in the General Population

**DOI:** 10.1038/s41598-018-37137-x

**Published:** 2019-03-14

**Authors:** Naoko Sawada, Masao Daimon, Takayuki Kawata, Tomoko Nakao, Koichi Kimura, Koki Nakanishi, Makoto Kurano, Megumi Hirokawa, Boqing Xu, Yuko Yamanaka, Tomoko S. Kato, Masafumi Watanabe, Yutaka Yatomi, Issei Komuro

**Affiliations:** 1 Department of Cardiovascular Medicine, The University of Tokyo Hospital, Tokyo, Japan; 2 Department of Clinical Laboratory, The University of Tokyo Hospital, Tokyo, Japan; 3 Department of Cardiovascular Medicine, Jichi Medical University, Tochigi, Japan; 4 Department of Cardiology, The Tokyo Metropolitan Geriatric Hospital and Institute of Gerontology, Tokyo, Japan

## Abstract

We evaluated the association between visceral adiposity and left ventricular (LV) diastolic function in association with plasma adiponectin levels in 213 subjects without overt cardiac diseases. Abdominal visceral fat area was quantified by computed tomography. Excessive visceral fat was significantly associated with impaired diastolic parameters including E/A, E′ and E/E′. Although serum adiponectin levels decreased with increased visceral adiposity, there was no independent association between serum adiponectin levels and diastolic parameters, which suggest that the role of adiponectin in this association might be indirect.

## Introduction

The number of heart failure patients with preserved ejection fraction (HFpEF) is continuously increasing and now accounts for nearly half of all HF patients^1,2^. Previous studies have shown that the most common cause of HFpEF is left ventricular (LV) diastolic dysfunction^3^, and the progression of diastolic dysfunction is an independent predictor of HFpEF^4^. LV diastolic function slowly declines with aging^5,6^, and is accelerated by diabetes mellitus, hypertension, hyperlipidemia, obesity and other clinical factors^7^. To reduce the incidence of future HFpEF, it is essential to understand the pathophysiology of progression of LV dysfunction and to prevent its progression.

Obesity is a major risk factor for the development of heart failure^8^, and is also an independent risk factor for LV diastolic dysfunction^9,10^. Although the mechanistic link between obesity and LV diastolic dysfunction is still debated, visceral adiposity has gained attention as one of the possible mechanisms for this link. A few studies reported that visceral adiposity assessed by computed tomography (CT) was associated with LV diastolic dysfunction in the general population^11,12^, patients with type 2 diabetic mellitus^13^, patients on peritoneal dialysis^14^ and patients with myocardial infarction^15^. In patients with type 2 diabetes mellitus, it was suggested that excessive visceral fat has a stronger association with the development of LV diastolic dysfunction than glycemic control^13^. However, the relationship between visceral adiposity and LV function has not been well defined in comparison with other clinical factors such as blood pressure, age or body weight in the general population.

In addition, the pathophysiological mechanism responsible for the relationship between visceral adiposity and LV diastolic function is not clear. Adiponectin, one of the adipocytokines produced by adipose tissue^16^, was reported to have cardioprotective effects^17^. Thus, some investigators suggested that adiponectin might serve as a therapeutic target in LV diastolic dysfunction^18^. Indeed, it was reported that plasma adiponectin levels were decreased in the general population according to the severity of visceral adiposity quantified by CT^19^. However, none of the previous studies that reported an association between visceral fat and LV diastolic function^11–15^ examined plasma adiponectin levels. Furthermore, some previous studies reported an inconsistent association between plasma adiponectin levels and LV diastolic function in selected populations^20–24^. Thus, it is still unclear if adiponectin is involved in the relationship between visceral fat and LV diastolic function.

The aim of our study was to elucidate the significance of the association between visceral adiposity and LV diastolic function in comparison with other clinical factors, and to determine if low plasma adiponectin levels or other metabolic parameters are involved in the association between visceral adiposity and LV diastolic function in the general population.

## Results

**Figure 1:**
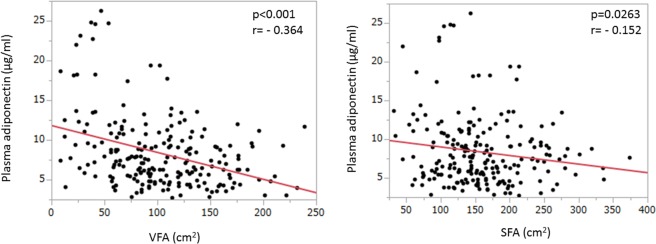
Correlation between VFA (visceral fat area), SFA (subcutaneous fat area) and plasma adiponectin concentration. Both VFA and SFA were statistically significantly correlated with plasma adiponectin levels, although VFA tended to have stronger association than SFA.

**Figure 2:**
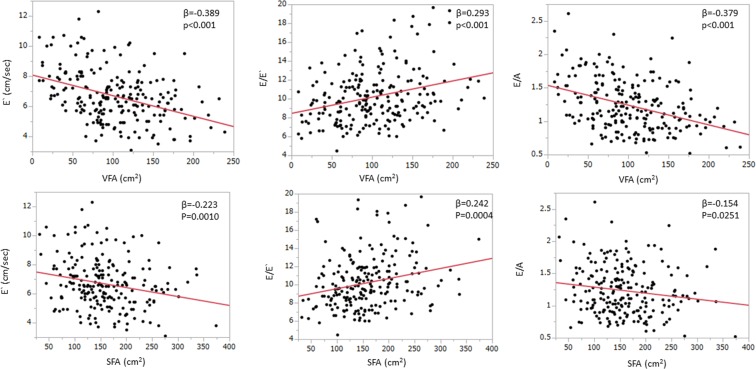
Regression plots showing correlation between VFA (visceral fat area), SFA (subcutaneous fat area) and diastolic parameters (E′, E/E′, E/A). VFA had significant correlations with all of these diastolic parameters and was also an independent determinant of them. SFA also showed the significant association with diastolic parameters like that obtained with VFA, although the associations were relatively weaker compared to those of VFA.

**Figure 3:**
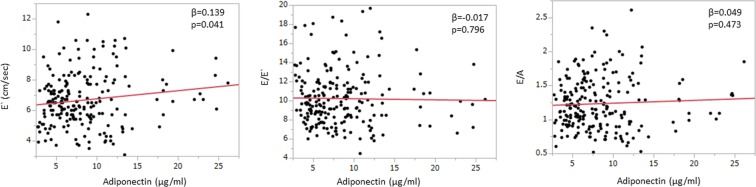
Correlation between adiponectin and echo parameters (E′, E/E′, E/A). Plasma adiponectin was mildly correlated with E′, but not with E/E′ and E/A.

A total of 213 participants (153 men, mean age 56 years) with preserved LV ejection fraction assessed by Teichholz formula were included in the study as shown in Table 1. Among these participants, 7% had diabetes mellitus, 5% had obesity and 25% had metabolic syndrome. The laboratory data, echocardiographic measurements and VFA measured by CT were also shown in Table 1. Plasma adiponectin was inversely correlated with VFA (Fig. 1). Table 2 shows correlations between the clinical factors related to metabolic syndrome and echocardiographic parameters. Age, WC, BMI, SBP, VFA and HbA1c strongly correlated with all diastolic parameters including E′, E/E′ and E/A. Although VFA shows the significant correlations with all diastolic parameters (Fig. 2), serum plasma adiponectin level had only a weak correlation with E′ (p = 0.041) and no significant correlation was observed between adiponectin level and E/E′ as well as E/A (Fig. 3). Subcutaneous fat area (SFA) also showed the significant association with plasma adiponectin level as well as diastolic parameters (Figs 1 and 2), although the associations were relatively weaker compared to those of VFA.

**Table 1:** Characteristics of the study population.

	Total n = 213	Men n = 153	Women n = 60	P value
Age, years	56 ± 10	56 ± 10	59 ± 10	0.004
Cardiovascular risk factors				
Hypertension, n (%)	42 (20)	31 (20)	11 (18)	0.750
Diabetes mellitus, n (%)	16 (7)	13 (8)	3 (5)	0.383
Dyslipidemia, n (%)	43 (20)	28 (18)	15 (25)	0.273
Obesity, n (%)	11 (5)	9 (6)	2 (3)	0.450
Metabolic syndrome, n (%)	55 (25)	44 (29)	11 (18)	0.118
Systolic blood pressure, mmHg	121 ± 14	122 ± 13	120 ± 16	0.259
Diastolic blood pressure, mmHg	77 ± 9	78 ± 8	76 ± 11	0.111
Heart rate, bpm	63 ± 10	63 ± 9	64 ± 10	0.359
BMI, kg/m^2^	24.0 ± 3.4	24.7 ± 3.2	22.2 ± 3.4	<0.0001
Waist circumference, cm	86.5 ± 8.9	88.4 ± 8.0	81.8 ± 9.6	<0.0001
Analytical data				
Total cholesterol, mg/dl	198 ± 36	193 ± 34	208 ± 38	0.0063
HDL-C, mg/dl	59 ± 17	55 ± 15	68 ± 18	<0.0001
LDL-C, mg/dl	121 ± 31	119 ± 30	127 ± 32	0.0971
Triglycerides, mg/dl	122 ± 92	132 ± 103	97 ± 50	0.0110
Creatinine, mg/dl	0.79 ± 0.17	0.85 ± 0.15	0.64 ± 0.14	<0.0001
Hemoglobin, mg/dl	14.1 ± 1.4	14.5 ± 1.2	12.9 ± 1.2	<0.0001
Hemoglobin A1c	5.8 ± 0.6	5.8 ± 0.6	5.8 ± 0.6	0.935
HOMA-IR score	1.35 ± 1.73	1.53 ± 1.92	0.89 ± 0.96	0.0151
Adiponectin, μg/ml	8.4 ± 4.5	6.9 ± 2.7	12.2 ± 5.8	<0.0001
Echocardiography				
LV mass index, g/m^2^	67.5 ± 13.9	70.0 ± 13.6	61.2 ± 12.6	<0.0001
LA volume index, ml/m^2^	27.2 ± 6.9	27.1 ± 6.4	27.5 ± 8.3	0.733
Ejection fraction, %	65 ± 5	64 ± 5	67 ± 4	0.0005
E wave, cm/s	65.1 ± 12.4	62.9 ± 10.5	70.8 ± 15.0	<0.0001
A wave, cm/s	56.5 ± 16.2	53.7 ± 15.4	63.4 ± 16.0	<0.0001
E/A ratio	1.24 ± 0.38	1.25 ± 0.36	1.20 ± 0.43	0.355
Deceleration time, ms	218 ± 39	217 ± 40	220 ± 39	0.640
E′ velocity, cm/s	6.7 ± 1.7	6.7 ± 1.6	6.7 ± 1.9	0.898
E/E′ ratio	10.2 ± 2.8	9.9 ± 2.9	11.0 ± 2.7	0.0084
Fat areas determined by CT scan				
Visceral fat area, cm^2^	102 ± 49	111 ± 47	80 ± 46	<0.0001
Subcutaneous fat area, cm^2^	159 ± 62	155 ± 60	167 ± 67	0.197

BMI, body mass index; HDL-C, high-density lipoprotein cholesterol; LDL-C, low-density lipoprotein-cholesterol; HOMA-IR, homeostasis model assessment of insulin resistance; LV, left ventricular; LA, left atrial; CT, computed tomography

Data are expressed as the mean ± SD or number (percentage).

**Table 2:** Pearson’s correlation between the echocardiographic parameters and clinical data.

	E′		E/E′		E/A		LA volume index		LV mass index	
	β	P	β	P	β	P	β	P	β	P
Age, year	−0.435	<0.001	0.462	<0.001	−0.421	<0.001	0.184	0.007	−0.063	0.355
WC, cm	−0.341	<0.001	0.270	0.001	−0.259	<0.001	0.030	0.658	0.152	0.026
SBP, mmHg	−0.344	<0.001	0.285	<0.001	−0.347	<0.001	0.117	0.086	0.300	<0.001
BMI, kg/m^2^	−0.305	<0.001	0.280	<0.001	−0.229	<0.001	0.050	0.461	0.204	0.003
VFA, cm^2^	−0.389	<0.001	0.293	<0.001	−0.379	<0.001	−0.027	0.686	0.026	0.696
HbA1c	−0.331	<0.001	0.271	<0.001	−0.289	<0.001	−0.004	0.944	−0.065	0.343
HOMA-IR	−0.153	0.025	0.112	0.103	−0.163	0.017	−0.044	0.515	0.001	0.991
Adiponectin, μg/ml	0.139	0.041	−0.017	0.796	0.049	0.473	0.062	0.367	−0.174	0.011
Creatinine, mg/dl	−0.128	0.060	−0.025	0.712	−0.029	0.664	−0.071	0.297	0.051	0.457
HDL-C, mg/dl	0.160	0.019	−0.075	0.272	0.085	0.216	−0.007	0.918	−0.050	0.465
Triglyceride, mg/dl	−0.139	0.043	0.061	0.373	−0.148	0.030	−0.095	0.164	0.046	0.498
Hemoglobin, mg/dl	−0.150	0.029	−0.050	0.466	−0.136	0.047	−0.232	0.001	0.034	0.618

WC, waist circumference; SBP. Systolic blood pressure; BMI, body mass index; VFA, visceral fat area; HbA1c, glycosylated hemoglobin; HOMA-IR, homeostasis model assessment of insulin resistance; HDL-C, high-density lipoprotein cholesterol.

Multivariable linear regression analyses demonstrated that age, SBP, and VFA were independently associated with all diastolic parameters including E′, E/E′ and E/A, whereas plasma adiponectin levels were not associated with any of diastolic parameters (Table 3).

**Table 3:** Multivariable linear regression analysis for diastolic parameters.

	E′		E/E′		E/A	
	β	P	β	P	β	P
Age, year	−0.372	<0.001	0.363	<0.001	−0.352	<0.001
VFA, cm^2^	−0.167	0.025	0.159	0.012	−0.170	0.019
SBP, mmHg	−0.169	0.006	0.151	0.016	−0.186	0.003
HbA1c	−0.169	0.013	0.110	0.082	−0.115	0.094
HOMA-IR	0.043	0.514			0.0011	0.986
Adiponectin, μg/ml	0.063	0.358				
HDL-C, mg/dl	−0.025	0.714				
Triglyceride, mg/dl	−0.046	0.508			−0.049	0.460
Hemoglobin, mg/dl	−0.130	0.059			−0.124	0.069

SBP. Systolic blood pressure; VFA, visceral fat area; HbA1c, glycosylated hemoglobin A1c; HOMA-IR, homeostasis model assessment of insulin resistance; HDL-C, high-density lipoprotein cholesterol.

ICC of inter observer variability for each measurement was LV mass index (0.79, [95% confidence interval: 0.39–0.94]), LA volume index (0.99 [0.95–0.997]), and E/E′ (0.91 [0.70–0.98]), respectively. ICC of intra-observer variability was LV mass index (0.84 [0.51–0.96]), LA volume index (0.99 [0.96–0.99]), and E/E′ (0.97 [0.90–0.99]), respectively.

## Discussion

In this study, we found that visceral adiposity as well as hypertension and age were independent and important factors for LV diastolic function in the general population. Although plasma adiponectin levels significantly decreased with increased visceral adiposity, the association of adiponectin level and diastolic function was not independent. Decreased plasma adiponectin does not appear to play a central role in the association between visceral adiposity and LV diastolic dysfunction. To the best of our knowledge, this is the first study to evaluate the independent relationships among visceral adiposity, plasma adiponectin levels and LV diastolic function, by measuring all of these parameters in the same general population with preserved LV ejection fraction.

Although the treatment of heart failure with reduced ejection fraction has improved drastically in the past two decades, no convincing treatment for HFpEF has been established^1,2^. Diastolic dysfunction is the most common cause of HFpEF^3^, and the progression of LV diastolic dysfunction is an independent predictor of HFpEF^4^. Given that the prognosis of patients with HFpEF has not improved in the past two decades^25,26^, prevention of the progression of LV diastolic function is the current best strategy to reduce the incidence of HFpEF. In this study, we found that visceral adiposity was an important and independent factor associated with LV diastolic dysfunction as well as SBP and age. Although some previous studies^11–15^ reported a significant relationship between visceral adiposity and LV diastolic function, the relationship between visceral adiposity and LV diastolic function may not be independent of other clinical factors. Our results suggest that controlling visceral adiposity as well as SBP might be important for preventing the progression of LV diastolic dysfunction with aging.

The pathophysiological mechanism underling the relationship between visceral adiposity and diastolic function is still uncertain. Adipose tissue secretes a number of biologically active adipokines such as adiponectin, leptin, resistin, tumor necrosis factor-α and interleukin-6^16^. Unlike other adipokines, adiponectin appears to be cardioprotective with both anti-inflammatory and anti-atherosclerogenic effects^17^; and decreased adiponectin levels are associated with increased cardiovascular risk factors such as low HDL-C, increased BMI, high fasting glucose and hypertension^27^. Low plasma adiponectin levels are known to be associated with insulin resistance and visceral rather than subcutaneous fat accumulation^28^. Some studies also demonstrated that low adiponectin levels may contribute to increased myocardial hypertrophy, cardiac fibrosis, nitrative and oxidative stress, angiogenesis, atherosclerosis and an inflammatory response through a number of adiponectin-mediated signaling pathways^18^. Furthermore, Ouchi *et al*.^29^ showed that low plasma adiponectin was associated with impaired endothelium-dependent vasorelaxation, which is closely associated with LV diastolic function^7^. Therefore, some investigators believe that adiponectin could be a therapeutic target in LV diastolic dysfunction^18^. However, there are only few clinical studies that examined the cardioprotective effect of adiponectin, which was reported to be decreased with increased visceral adiposity^19^, and the number of clinical evidences that prove the association between adiponectin and LV diastolic function is insufficient.

Some previous investigations reported an inconsistent association between plasma adiponectin and LV diastolic function^20–24^. A few studies showed a positive correlation between plasma adiponectin and LV diastolic dysfunction in conditions such as hypertension^20^, hypertrophic cardiomyopathy^21^ or early LV diastolic dysfunction^22^. However, some consideration should be given to interpretation of these results. Although Hong *et al*.^20^ found a significant relationship between plasma adiponectin and LV diastolic function assessed only by mitral inflow parameters in univariate analysis, they did not report if the association was independent of other clinical variables in multivariate analysis. Unno *et al*.^21^ investigated this relationship in 26 patients with hypertrophic cardiomyopathy, but statistical significance in such a small population might be weak and the pathological link between a genetic disease of the cardiac sarcomeres and plasma adiponectin levels is uncertain. Similarly, Negi *et al*. reported a significant relationship between low plasma adiponectin levels and diastolic dysfunction in hypertension^22^, but their study included only 25 cases with hypertension and 25 age-matched controls. In contrast, there are other studies which did not find a significant relationships between plasma adiponectin levels and diastolic function in type 2 diabetes mellitus^23^, or in the general population^24^. As for the effect of adiponectin on LV hypertrophy, we found a significant association in this study, and this confirms the results of a previous large-scale study^30^. However, visceral adipose tissue is a multifunctional organ, which secretes not only adiponectin, but also a number of biologically active adipokines^16^. We may conclude that adiponectin does not have a significant effect on the link between visceral adiposity and LV diastolic dysfunction. We need further studies to determine the precise mechanisms underlying this link. Interestingly, a recent study from Fontes-Carvalho *et al*.^24^ demonstrated that leptin rather than adiponectin was associated with LV diastolic dysfunction, although they did not assess visceral adiposity. Regrettably, we did not measure plasma leptin levels in the current study.

On the other hand, apelin is an endogenous peptide which belong to the family of adipocytokines. Apelin has an increasingly recognized role in cardiovascular regulation in both animals and humans^31^. Experimental study demonstrated that apelin-knockout mice showed progressive and significant left ventricular dilatation and systolic dysfunction, which was attenuated by subcutaneous apelin infusion^32^. Zhang *et al*. showed that apelin is a negative regulator of angiotensin II-mediated adverse myocardial remodeling and dysfunction in a murine model^33^. Clinical studies also demonstrated decreased serum and myocardial apelin levels in patients with heart failure^34,35^. Furthermore, Boal F *et al*. demonstrated that obese heart failure patients have greater levels of plasma apelin than non-obese heart failure patients, which might provide the mechanism basis of “obesity paradox”^36^. Taken together, measurement of apelin level will provide valuable information to elucidate the underlying mechanism between obesity and diastolic dysfunction as well as to explore therapeutic strategies for diastolic dysfunction. Future study is warranted to investigate the association of serum apelin levels with LV diastolic dysfunction in larger number of general population.

Several limitations of present study should be considered. First, because the study sample was relatively young and few individuals were obese, the observed association might not allow generalization to populations with different demographic composition especially obese subjects. Second, we did not have LVEF measurement by Simpson’s methods. Although we exclude the participants with LV wall motion abnormalities, LVEF measurements by Simpson’s methods is more ideal than measurement by Teichholz formula^37^. In addition, the interpretation of E/E′ might be overestimates because there is a possible variability of E/E′ between the measure on the medial annulus and lateral wall. Unfortunately, our database did not contain the measurement of E/E′ on the lateral wall, although current guideline recommends the use of average of the two^38^. Third, the study population had normal or grade 1 diastolic function, and subjects with more than moderate LV diastolic dysfunction were not included. A larger-scale study including patients with moderate or severe LV diastolic dysfunction is needed to confirm our results. Forth, although apelin emerges as a novel biomarker against cardiovascular diseases which is a member of the adipose tissue-derived peptides, we did not measure them. Future studies is needed to evaluate the association between serum apelin levels and diastolic function to elucidate the underlying mechanism regarding obesity-related diastolic dysfunction. Finally, this was a cross-sectional observational study. Further longitudinal and interventional studies are required to verify if visceral adiposity could be a therapeutic target for preventing progression of LV diastolic dysfunction.

## Methods

### Study population

This study had a cross-sectional design. Initially we enrolled 215 constructive subjects who underwent laboratory test, 2-dimensional echocardiography, and abdominal CT in the health check-up clinic at the University of Tokyo Hospital between April 2016 and June 2016. The exclusion criteria were: (1) LV ejection fraction <50%; (2) atrial fibrillation; (3) LV regional wall motion abnormalities; (4) significant valvular disease (more than mild grade), (5) cardiomyopathy (6) history of coronary artery disease and (7) congenital heart disease. Among all, 2 participants met exclusion criteria (1 for were coronary artery disease and 1 for congenital heart disease) and were excluded. Thus, the final population of this study comprised 213 participants (153 men, mean age 56 years). This observational study was approved by the institutional ethics committee of the University of Tokyo (#2650) and did not involve any of experiments. Written comprehensive informed consent that allows all de-identified data to be used for any study including our research protocol was obtained from all participants at the time of health check-up. We confirm that all methods were carried out in accordance with relevant guidelines and regulations.

### Measurement of clinical parameters

The medical health checkup included the following items: height, body weight, waist circumference (WC), systolic blood pressure (SBP), diastolic blood pressure (DBP), blood-cell counts, blood chemistry, electrocardiography, chest radiography, transthoracic echocardiography and abdominal CT. Body mass index (BMI) was calculated as body weight (kg) divided by height-squared (m^2^).

### Definitions of clinical variables

Hypertension was defined as SBP ≥140 mm Hg or DBP ≥90 mm Hg at the time of visit or use of antihypertensive drugs. Diabetes was defined as glycosylated hemoglobin A1c (HbA1c) ≥6.5% or the use of antidiabetic drugs. Dyslipidemia was defined as low-density lipoprotein-cholesterol (LDL-C) ≥140 mg/dl or high-density lipoprotein cholesterol (HDL-C) <40 md/dl or triglyceride ≥150 mg/dl or the use of lipid lowering drugs. We defined obesity (BMI ≥30) according to the International Classification of adult using BMI^39^. Metabolic syndrome was diagnosed if any of the following conditions were present: central obesity (WC ≥85 cm in men and WC ≥90 cm in women), dyslipidemia (triglyceride ≥150 mg/dl or/and HDL-C <40 mg/dl), raised blood pressure (SBP ≥130 mmHg or/and DBP ≥85 mmHg) or a disorder of glycometabolism (HbA1c ≥6.5% or previously diagnosed diabetes) based on the Japanese guideline^40^.

### Measurement of laboratory parameters

Venous blood samples were collected between 8:00 and 10:00, after 12 hours overnight fast, with the participant in sitting position. Serum total cholesterol, LDL-C, HDL-C, triglyceride, hemoglobin, creatinine, and HbA1c by immunonephelometry. For insulin measurement, the blood was immediately centrifuged and the plasma stored at −20 °C for later analysis. Insulin resistance was assessed using the homeostasis model assessment of insulin resistance (HOMA-IR) score. The HOMA-IR score was calculated from the following formula:

$${\rm{HOMA}}-{\rm{IR}}={\rm{fasting}}\,{\rm{glucose}}\,(\mathrm{mg}/\mathrm{dl})\times {\rm{insulin}}\,({\rm{\mu }}{\rm{U}}/\mathrm{ml})/405$$

Serum total adiponectin levels were measured by an enzyme-linked immunosorbent assay using the Human Adiponectin Latex Kit (Eiken Chemical Co., Ltd., Tokyo, Japan), using an automatic clinical chemistry analyzer, Labospect008 Hitachi LABOSPECT 008 (Hitachi High-Tech Co., Tokyo, Japan). When we investigated within-run precision of this method, measuring a pooled human serum sample for twenty times, we observed that the mean ± SD for the within-run study were 12.67 ± 0.08 g/mL and that the within-run CVs were 0.63%.

### Echocardiographic imaging

Echocardiographic examination was performed using commercially available system (Toshiba Aplio, Toshiba Medical System Corp, Tochigi, Japan). All images were acquired in the left lateral decubitus position according to a standardized protocol and interpreted by a trained imaging cardiologist who was blinded to all other clinical information. The echocardiographic equipment was maintained according to the guideline of the Japanese Society of Echocardiography^41^. Echocardiographic images were obtained using commercially available systems from the parasternal, apical and subcostal windows for the evaluation of right and left ventricular function. Two-dimensional and color Doppler imaging were performed to screen for valvular stenosis or regurgitation. LV and left atrial (LA) chamber quantifications were performed with 2-dimensional echocardiography, according to the guidelines of the American Society of Echocardiography^37^. LV ejection fraction was calculated using the Teichholz formula. LV mass was calculated by a validated Devereux’s formula^42^ using LV diastolic diameter and wall thickness on 2-dimensional echocardiography, and was indexed by body surface area. LA volume was measured using the biplane area-length method and was calculated as LA volume/body surface area. We evaluated diastolic dysfunction as previously described^9,38^. Briefly, peak velocities of the early (E-wave) and late (A-wave) phases of the mitral inflow pattern from Doppler recordings were measured in the apical four-chamber view, and their ratio (E/A) was calculated. The peak early diastolic (E′) velocities of the septal mitral annulus were measured by pulsed tissue Doppler imaging in the apical four-chamber view. The ratio between the E and the E′ wave (E/E′) was then calculated. To estimate inter- and intra-reader variability, two independent observers performed echocardiographic measurements of LV mass index, LA volume index and E/E in randomly selected 10 subjects and one observer repeated the measurements subsequently. The inter- and intra-observer variability was estimated using an intra-class correlation coefficients (ICC).

### Assessment of visceral adiposity by CT

Visceral adiposity was assessed as visceral fat area (VFA) quantitatively measured by CT taken at the level of the umbilicus as previously reported^43^. The area of visceral fat was defined as the sum of the intraperitoneal fat area with CT density in the range of −150 to −50 Hounsfield units. Subcutaneous fat area was also measured in the same manner.

The datasets generated during and/or analyzed during the current study are available from the corresponding author on reasonable request.

### Statistical analysis

Statistical analyses were performed using JMP version Pro 11. Data are expressed as the mean ± standard deviation for quantitative variables with normal distribution. The relationships between variables were assessed using Pearson’s correlation coefficient. Univariable and multivariable linear regression analyses on diastolic parameters were performed to identify clinical and laboratory variables that were associated with diastolic parameters. The factors related at the p < 0.05 level were selected as independent variables for multivariable analysis. All tests of significance were 2-tailed, and P < 0.05 was considered statistically significant.
